# Effectiveness of Chinese herbal medicine on improving sleep and mental health in adults: Study protocol for a stepped-wedge cluster randomized trial

**DOI:** 10.12688/f1000research.163698.1

**Published:** 2025-06-04

**Authors:** Hiu To Tang, Hoi Ki Wong, Jingyuan Luo, Jialing Zhang, Qin Liu, Lixiang Zhai, Rachel Ngan Yin Chan, Yun Kwok Wing, Shirley Xin Li, Wing Fai Yeung, Albert Yeung, Francesco Recchia, Chun Hoi Cheung, Junjun Yang, Danny J. Yu, Zhaoxiang Bian

**Affiliations:** 1 Vincent V.C. Woo Chinese Medicine Clinical Research Institute, School of Chinese Medicine, Hong Kong Baptist University, Hong Kong SAR, China; 2 School of Chinese Medicine, Hong Kong Baptist University, Hong Kong SAR, China; 3 Centre for Chinese Herbal Medicine Drug Development Limited, Hong Kong Baptist University, Hong Kong SAR, China; 4 Li Chiu Kong Family Sleep Assessment Unit, Department of Psychiatry, Faculty of Medicine, The Chinese University of Hong Kong, Hong Kong SAR, China; 5 Department of Psychiatry, Faculty of Medicine, The Chinese University of Hong Kong, Hong Kong SAR, China; 6 Sleep Research Clinic and Laboratory, Department of Psychology, The University of Hong Kong, Hong Kong SAR, China; 7 The State Key Laboratory of Brain and Cognitive Sciences, The University of Hong Kong, Hong Kong SAR, China; 8 School of Nursing, The Hong Kong Polytechnic University, Hong Kong SAR, China; 9 Depression Clinical and Research Program, Massachusetts General Hospital, Boston, USA; 10 School of Public Health, Li Ka Shing Faculty of Medicine, The University of Hong Kong, Hong Kong SAR, China; 11 The Chinese Medicine Hospital of Hong Kong, Hong Kong SAR, China

**Keywords:** Chinese herbal medicine, depression, anxiety, insomnia, stepped-wedge trial, stepped-care model

## Abstract

**Background:**

Depression, anxiety, and insomnia significantly impact global health and impose substantial health burdens worldwide. Conventional treatments including pharmacotherapy and psychotherapy face limitations in efficacy, side effects, and accessibility. Despite preliminary evidence suggesting the potential of Chinese herbal medicine (CHM) in managing these conditions, large-scale evaluations are lacking. This study aims to examine the effectiveness of CHM for the treatment of adults experiencing mild-to-moderate symptoms of depression, anxiety, and insomnia.

**Methods:**

This study will be a multicenter, assessor-blinded, pragmatic stepped-wedge cluster randomized trial. Districts will be randomized to receive the CHM intervention sequentially in four steps based on a computer-generated number, while control districts remain unexposed until their respective intervention phase. The intervention consists of a three-level stepped-care approach: Level 1 involves herbal tisane for mild symptoms, Level 2 offers standardized CHM for moderate symptoms or non-responders to Level 1, and Level 3 combines personalized CHM with psychotherapy for those not improved by Level 2. Symptom severity will be assessed using validated scales, Patient Health Questionnaire-9 for depression, Generalized Anxiety Disorder-7 for anxiety, and Insomnia Severity Index for insomnia, with primary outcomes measured at baseline, post-intervention, and follow-up. The study was approved by the Hong Kong Baptist University Research Ethics Committee (Approval No.: REC/22-23/0709).

**Discussion:**

This study will provide significant insights into the effectiveness of a stepped-care CHM intervention for alleviating depression, anxiety, and insomnia. The results could guide future decision-making regarding the integration of CHM into mainstream healthcare systems in Hong Kong and beyond.

**Trial registration:**

Chinese Clinical Trial Registry, ChiCTR2400083685, April 30, 2024, https://www.chictr.org.cn/hvshowprojectEN.html?id=251238&v=1.0

**Abbreviations**
AE: Adverse event
CHM: Chinese herbal medicine
DMC: Data monitoring committee
DSM-IV
: Diagnostic and Statistical Manual of Mental Disorders, fourth edition
GAD-7: General anxiety disorder-7
I-BMS
: Integrative body-mind-spirit
ICC: Intra-cluster correlation coefficient
ISI: Insomnia severity index
PHQ-9: Patient health questionnaire-9
PSS-10: Perceived Stress Scale
RCT: Randomized controlled trial
SF-12: 12-item short form survey
TCM: Traditional Chinese medicine

## 1. Introduction

### 1.1 Background and rationale

Mental and sleep disorders significantly impact global health, with depression, anxiety, and insomnia being the most prevalent and debilitating conditions. ^1^ Epidemiological studies indicate that depression is a leading cause of suicide, ^2^ while anxiety is the most common mental disorder across the globe. ^3^ Insomnia, often comorbid with both depression and anxiety, affects up to a third of the adult population worldwide. ^4^ The consequences of depression, anxiety, and insomnia extend beyond psychological health, exerting a drastic influence on physical well-being. Extensive research has indicated associations between the disorders and increased risks of developing physical comorbidities such as medically unexplained somatic symptoms, ^5^ cardiovascular diseases, ^6– 8^ diabetes, ^9– 11^ and cancer. ^12, 13^ Moreover, the global burden of mental disorders is escalating. According to the Global Burden of Diseases, Injuries, and Risk Factors Study of 2019, ^14^ depressive and anxiety disorders were the two most disabling mental disorders, both ranked among the top 25 leading causes of burden worldwide. ^15^ The pervasive impact of depression, anxiety, and insomnia present an urgent and nonnegligible challenge on the healthcare system.

According to clinical practice guidelines, pharmacotherapy and psychotherapy are the commonly recommended treatments for depression, anxiety and insomnia. ^16– 19^ However, recent evidence suggests that the efficacy of both treatments may have been overestimated. ^20^ Furthermore, pharmacotherapy presents major limitations, including delayed effects, non-responsiveness, risk of addiction, tolerance after long-term usage, and side effects. ^21– 23^ Psychotherapy can instead be limited by the unavailability of trained therapists and the high costs. ^24^ This is insufficient to combat the growing prevalence of mental and sleep disorders, especially in areas with a scarcity of mental health professionals. In Hong Kong, patients may need to wait for over two years for psychiatric consultations which last 6-8 minutes in average. ^25^ Moreover, up to three quarters of individuals suffering from mental disorders never seek medical help, ^26^ likely due to the social stigma and potential side effects attached to clinical treatments. ^27– 29^ Therefore, it is imperative to explore alternative treatments that are effective, accessible, and acceptable to meet the increasing needs of mental health care in the community.

Traditional Chinese medicine (TCM), including Chinese herbal medicine (CHM), acupuncture, dietary advice and exercise, etc., has been practiced for thousands of years for the treatment of depression, anxiety, and insomnia. Previous studies have reported the benefits of CHM in managing these conditions. For example, the widely used Xiao Yao decoction has been shown to elicit comparable benefits as antidepressants in alleviating depressive symptoms. ^30^ A recent meta-analysis reported that Xiao Yao decoction also improved insomnia symptoms. ^31^ Similarly, two randomized controlled trials (RCTs) have demonstrated the positive effects of Gui Pi and Huanglian Ejiao decoctions on anxiety symptoms. ^32, 33^ Importantly, patients treated with CHM were reported with fewer side effects compared to those taking antidepressants, anxiolytics, and sedatives. ^30– 34^ Furthermore, the holistic approach of TCM has been reported to be associated with less social stigma when compared to conventional psychological and psychiatric services. ^35^ This is particularly important as it could encourage more individuals to seek medical care without fear of being labelled or judged. Therefore, the implementation of CHM into conventional primary care settings may serve to promote help-seeking behavior from patients, while offering promising therapeutic benefits as well as reducing the burden of mental disorders on the healthcare system.

Although the effectiveness of CHM in alleviating sleep and mood disturbances has been previously reported, few studies examined the effectiveness of a stepped-care model for delivering CHM interventions. Evaluating such a model through traditional parallel randomized controlled trials (RCTs) could be limited by potential ethical concerns. ^36^ Instead, the stepped-wedge design presents a practical alternative for the evaluation, where the intervention is sequentially implemented across various time periods in a randomized manner. ^37^ This method has been successfully applied in various settings, including evaluating a sleep intervention using mentored behavioral and environmental restructuring to enhance sleep, ^38^ and a non-specialist delivered mental health intervention to improve common mental disorders and quality of life. ^39^ The stepped-wedge design not only facilitates continuous data collection at each intervention phase but also addresses ethical considerations by ensuring that all participants eventually receive the presumed beneficial intervention. ^36^ Additionally, this design allows for effective resource management when simultaneous intervention delivery is unfeasible and provides substantial analytical benefits by enabling participants to serve as their own controls, thus enhancing the ability to assess the impact of time on intervention efficacy. ^36^ Despite these benefits, the stepped-wedge design has not yet been applied to test the effectiveness of a stepped-care CHM intervention for depression, anxiety and insomnia.

### 1.2 Objectives

To address this gap, the current study employs a stepped-wedge cluster randomized trial design to evaluate the effectiveness of a stepped-care CHM intervention for the treatment of adults experiencing mild-to-moderate symptoms of depression, anxiety, and insomnia.

## 2. Methods

### 2.1 Trial design

**Figure 1.**
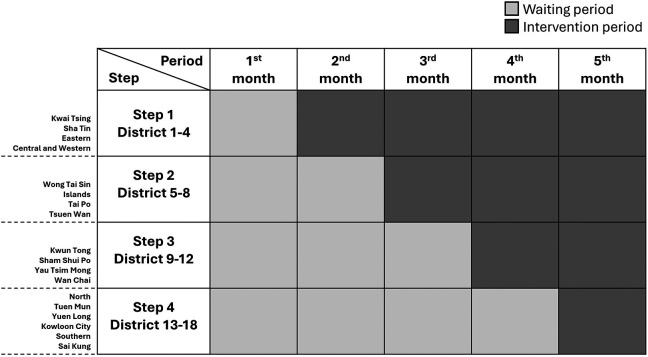
Stepped-wedge study design. Note: This study will encompass 18 geographic districts in Hong Kong, with randomization occurring at the district level. The intervention will be rolled out sequentially to 4-6 districts at a time, based on a computer-generated random number. Meanwhile, the remaining districts will remain unexposed during the control period. After four steps of exposure, all districts will receive the intervention. Light grey boxes indicate the control period, while dark grey boxes represent the intervention period.

This interventional study will be a multicenter, pragmatic, stepped-wedge, cluster randomized trial. Randomization will be carried out based on the 18 geographic districts in Hong Kong. The CHM intervention will be sequentially implemented in 4-6 districts per step according to a computer-generated random number while the remaining clusters will continue to stay unexposed to the intervention. After a 4-step exposure, all districts will receive the CHM intervention ( Figure 1). The study was approved by the Hong Kong Baptist University Research Ethics Committee (Approval No.: REC/22-23/0709) on April 8, 2024, and prospectively registered with the Chinese Clinical Trial Registry (ChiCTR2400083685) on April 30, 2024, https://www.chictr.org.cn/hvshowprojectEN.html?id=251238&v=1.0. Recruitment started on May 1, 2024, and has already been completed. Data collection is expected to end in July 2025. The study is conducted according to the guidelines for good clinical practice (ICH/GCP) and the Declaration of Helsinki. ^40^ This protocol strictly adhered to the Standard Protocol Items: Recommendations for Interventional Trials (SPIRIT) 2013 statement ( **refer to data availability**).

### 2.2 Study setting

The study will be conducted at three clinical centers, namely the Haven of Hope Hong Kong Baptist University Chinese Medicine Specialty Clinic, the Jockey Club Chinese Medicine Disease Prevention and Health Management Centre, and the Hong Kong Baptist University Mr. & Mrs. Chan Hon Yin Chinese Medicine Specialty Clinic and Good Clinical Practice Centre.

### 2.3 Eligibility criteria

**Inclusion criteria:** 1) Aged between 18 and 65 years; 2) Experiencing mild-to-moderate depression, anxiety, and insomnia, defined as a Patient Health Questionnaire-9 (PHQ-9) score between 5 and 19, a General Anxiety Disorder-7 (GAD-7) score between 5 and 14, or an Insomnia Severity Index (ISI) score between 8 and 21; 3) Understand and be able to follow written and oral instructions in Chinese to complete the assessment questionnaires; 4) Willing and able to provide informed consent.

**Exclusion criteria:** 1) History of alcohol and/or substance abuse disorder; 2) history of other major psychiatric or psychotic disorders (e.g., mania, schizophrenia, bipolar disorder, post-traumatic stress disorder or obsessive-compulsive disorder); 3) History of major neurologic disorder (e.g., Parkinson disease, Alzheimer disease or other dementia, brain tumor, seizure disorder or delirium episode); 4) Current active suicidal or self-injurious potential necessitating immediate treatment, or score ≥1 on the 9th item (suicidal ideation) in PHQ-9; 5) History of serious chronic illness, such as cancer, stroke, heart diseases, end-stage liver and kidney diseases; 6) Pregnant or lactating women; 7) Currently undergoing prescribed psychotropic medication, psychotherapy or TCM treatments for sleep and mood disturbances; 8) Allergic to CHM; and 9) Concurrent participation in other clinical trials.

### 2.4 Informed consent

Before participation, verbal and written information on the study will be provided. All individuals will undergo a detailed informed consent process where they are informed about the study’s procedures, risks, and benefits, ensuring their right to withdraw at any time. Written informed consent will be obtained before baseline outcome assessments.

### 2.5 Interventions

**2.5.1 Explanation for the choice of comparators**

The CHM intervention will be sequentially rolled out in a stepped wedge study design. In this design, each cluster contributes observations from both the unexposed (control) and the exposed (intervention) periods. This allows each cluster to serve as its own comparator over time. Additionally, comparisons are made between different clusters that receive the intervention at staggered times. This method ensures that every cluster acts as both a control and an intervention group, facilitating robust intra- and inter-cluster comparisons to assess the effectiveness of the CHM intervention.

**2.5.2 Intervention description**

**Figure 2.**
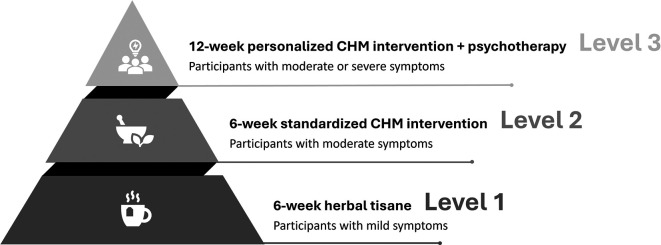
Stepped-care intervention. Note: Mild symptoms: a PHQ-9 score ranging from 5 to 9, a GAD-7 score from 5 to 9, or an ISI score from 8 to 14; Moderate symptoms: a PHQ-9 score ranging from 10 to 19, a GAD-7 score from 10 to 14, or an ISI score from 15 to 21; Severe symptoms: a PHQ-9 score ranging from 20 to 27, a GAD-7 score from 15 to 21, or an ISI score from 22 to 28.

In stepped-care models, the initial approach involves starting patients with a low intensity intervention. Progress is systematically monitored, and patients who do not adequately respond to the intervention can advance to a subsequent treatment of higher intensity or incorporate an additional treatment. ^41^ The stepped-care model has been proven to yield better clinical outcomes, enhanced efficiency in terms of resource use and cost. ^42^ Therefore, a stepped-care model will be employed to deliver the CHM interventions in the present study ( Figure 2).

**Level 1: Herbal Tisane**

The Level 1 intervention, utilizing a low-intensity tisane including four herbs, is designed for participants with mild symptoms of depression, anxiety, and insomnia, defined by a PHQ-9 score ranging from 5 to 9, a GAD-7 score from 5 to 9, or an ISI score from 8 to 14. A Chinese medicine practitioner will prescribe the herbal tisane based on the participant's main complaints. The Level 1 program includes three types of herbal tisanes for depression, anxiety, and insomnia respectively. Details of these tisanes are listed in Table 1. The level 1 intervention will last for 6 weeks, where participants will be instructed to take the tisane twice daily. Upon completion of the six-week intervention, participants whose main complaints escalate to moderate level will be stepped up to receive the level 2 intervention, while other participants who present with mild/minimal symptoms will be followed up for another 6 weeks to examine the short-term effects of herbal tisane intervention.

**Table 1.** The composition of the herbal tisane (Level 1 intervention).

	Latin Pharmaceutical Name	Chinese name	g/sachet
Depression Tisane	Curcumae Radix	Yujin	2
	Paeoniae Radix Alba	Baishao	3
	Fructus Tritici Levis	Fuxiaomai	4
	Acanthopanacis Senticosi Radix et Rhizoma Sue Caulis	Ciwujia	3
Anxiety Tisane	Nelumbinis Plumula	Lianzixin	0.3
	Paeoniae Radix Alba	Baishao	2
	Margaritifera Concha	Zhenzhumu	5
	Fructus Tritici Levis	Fuxiaomai	4
Insomnia Tisane	Coptidis Rhizoma	Huanglian	0.6
	Cinnamomi Cortex	Rougui	0.3
	Margaritifera Concha	Zhenzhumu	5
	Poria cum Radix Pini	Fushen	3

**Level 2: Standardized CHM intervention**

The Level 2 intervention, employing a higher-intensity formula consisting of 5 to 11 herbs, is designed for participants experiencing moderate symptoms of depression, anxiety, and insomnia, defined by a PHQ-9 score ranging from 10 to 19, a GAD-7 score from 10 to 14, or an ISI score from 15 to 21. A Chinese medicine practitioner will prescribe the herbal formula based on the participant's TCM syndrome. The Level 2 intervention includes three types of herbal formulas for excess syndrome, deficiency syndrome and deficiency-excess complex syndrome, respectively. The classification of these three syndromes will primarily rely on the clinical judgment of the Chinese medicine practitioner, who will consider the patients' symptoms, signs, and other body constitutional characteristics. Importantly, all herbal formulas are standardized, which facilitates the evaluation of their effectiveness and allows for easy replication in future studies. Details of the three formulas are listed in Table 2. The level 2 CHM intervention will last for 6 weeks, where participants will be instructed to take the herbal formula twice a day. Participants whose symptoms do not downgrade to mild level in their assessment score after level 2 intervention will be stepped up to receive the level 3 intervention, while other participants who downgrade to mild/minimal symptoms will be followed up for another 6 weeks to examine the short-term effects of the herbal formula intervention.

**Table 2.** The composition of the herbal formulas (Level 2 intervention).

	Name	Ingredients	g/day
Excess syndrome	Jia Wei Xiao Yao San	Bupleuri Radix, Angelicae Sinensis Radix, Paeoniae Radix Alba, Atractylodis Macrocephalae Rhizoma, Poria, Glycyrrhizae Radix et Rhizoma, Moutan Cortex, Gardeniae Fructus	10
Deficiency syndrome	Gui Pi Tang	Ginseng Radix et Rhizoma, Jujubae Fructus, Ziziphi Spinosae Semen, Glycyrrhizae Radix et Rhizoma, Polygalae Radix, Zingiberis Rhizoma Recens, Angelicae Sinensis Radix, Atractylodis Macrocephalae Rhizoma, Poria cum Radix Pini, Astragali Radix, Longan Arillus	10
Deficiency-excess Complex syndrome	Huang Lian E Jiao Tang and Jiao Tai Wan	Coptidis Rhizoma, Scutellariae Radix, Paeoniae Radix Alba, Asini Corii Colla, Cinnamomi Cortex	10.3

**Level 3: Personalized CHM intervention combined with psychotherapy**

Level 3 intervention integrates an 8-week personalized CHM intervention and an 8-session psychotherapy. The CHM intervention will be modified based on the 3 herbal formulas employed in the Level 2 intervention, enhancing the personalization by adding up to 5 additional herbs to specifically address symptoms with the highest level of precision and intensity among the 3 levels of treatment. The psychotherapy will adopt an integrative body-mind-spirit (I-BMS) psycho-social approach, which emphasizes a holistic concept of health and recognizes the inter-connectedness between the body, mind and spirit. During the treatment, project social workers and counsellors will introduce I-BMS knowledge, mindfulness/body-mind physical exercises to enhance the mental health of the participants. The intervention aims to improve their mental well-being and self-management strategies through a combination of casework and groupwork approaches. The counselling treatment consists of eight sessions (1.5 hours each) which are expected to be completed within 12 weeks, followed by a 12-week post-intervention follow-up.

**2.5.3 Criteria for discontinuing or modifying allocated interventions**

All patients could voluntarily participate in and withdraw from the study at any time without any reason or consequences. The treatment will be discontinued if the patient encounter a serious adverse reaction.

**2.5.4 Strategies to improve adherence to interventions**

To improve adherence to the intervention, enrolled participants in Level 1 will be contacted bi-weekly, and those in Level 2 and 3 will be contacted weekly by phone as a reminder to take their medication. At the end of the intervention, participants will be required to return both the empty packets from consumed sachets and any sachets that remain unconsumed.

**2.5.5 Relevant concomitant care permitted or prohibited during the trial**

Participants will be advised to maintain their usual daily habits throughout the intervention, including their diet and physical activity levels.

### 2.6 Outcomes

**Primary outcomes**

a) **Depression**
  The Patient Health Questionnaire-9 (PHQ-9) instrument is a 9-item scale measuring different aspects and severity of depression. It asks participants how often they experienced depressive symptoms over the past 2 weeks. PHQ-9 comprises 5 categories: 0–4 indicates minimal depression, 5–9 mild depression, 10–14 moderate depression, 15–19 moderately severe depression, and ≥20 severe depression. In the present study, a PHQ-9 score of 10-19 (moderate and moderately severe depression in original classification) will be deemed moderate depression for easy administration. The Chinese version of PHQ-9 has been validated in Hong Kong adults with good reliability (Cronbach's alpha: 0.82). ^43^
b) **Anxiety**
  The General Anxiety Disorder-7 (GAD-7) instrument is a 7-item scale modified from the diagnostic criteria of Diagnostic and Statistical Manual of Mental Disorders, fourth edition (DSM-IV) for screening generalized anxiety disorder and severity. The cut-off scores of GAD-7 are: 0–4 indicates minimal anxiety, 5–9 mild anxiety, 10–14 moderate anxiety, and ≥15 severe anxiety. The Chinese version of GAD-7 has been validated in general hospital outpatients with a Cronbach's alpha of 0.90. ^44^
c) **Insomnia**
  The Insomnia Severity Index (ISI) includes seven items assessing: 1) sleep onset, 2) sleep maintenance difficulties, 3) satisfaction with the current sleep pattern, 4) interference with daily functioning, 5) noticeable impairment due to sleep problems, 6) degree of distress, and 7) concerns caused by sleep problems. A total score of 0–7 indicates “no clinically significant insomnia,” 8–14 suggests “subthreshold insomnia,” 15–21 is classified as moderate severity, and 22–28 indicates severe insomnia. The Chinese version of ISI has been shown a satisfactory content validity index of 0.94 and high internal consistency with a Cronbach’s alpha of 0.81. ^45^

**Secondary outcomes**

a) **Health-related quality of life**
  The 12-item short form survey (SF-12) measures health-related quality of life across 8 domains - physical functioning, role limitations due to physical health, body pain, general health perceptions, vitality, social functioning, role limitations due to emotional problems, and mental health. The Chinese version of SF-12 has been shown a good reliability with a Cronbach’s alpha of 0.828. ^46^
b) **Perceived stress**
  The Perceived Stress Scale (PSS-10) is a 10-item scale that measures an individual's perception of stress over the past month. It assesses the degree to which situations are perceived as stressful, unpredictable, uncontrollable and overloading. Six negative items are scored from 0 (never) to 4 (very often), and 4 positive items are reversely scored. Total scores range from 0 to 40, with higher scores indicating greater perceived stress. The Chinese version of PSS-10 showed a stable two-factor structure with a satisfactory internal consistency (Cronbach’s alphas = 0.67 to 0.78). ^47^
c) **Utilization of healthcare resources**
  A questionnaire will record the participants' help-seeking behaviors related to mental health and sleep issues over the past month, including: the types of healthcare professionals consulted, such as psychiatrists, psychologists, counselors, social workers, nurses, and general practitioners; the number of visits made to healthcare professionals; and the expenditures associated with help-seeking for mental health or sleep issues.
d) **TCM body constitution assessment**
  A comprehensive TCM symptom questionnaire will be administered to evaluate changes in symptoms from the TCM perspective. The questionnaire will assess symptoms across four TCM examinations, such as psychiatric symptoms (e.g. anxiety, depression, irritability), somatic symptoms (e.g. headache, dizziness, fatigue), digestive-related symptoms (e.g. appetite, abdominal pain, constipation, diarrhea), and sleep-related symptoms. The questionnaire will also include tongue and facial inspection, and pulse assessment. Each symptom will be rated by a registered Chinese medicine practitioner based on how often the participant experienced the symptoms over the past 2 weeks on a 4-point scale from 0 (not at all) to 3 (nearly every day).
e) **Compliance rate**
  The compliance rate, defined as the proportion of prescribed CHM sachets consumed by participants. Participants will be required to return both the empty packets of consumed sachets and any remaining unconsumed sachets after the end of intervention. Compliance rate will be calculated for each participant using the formula: (number of sachets consumed/total number of sachets prescribed) ×100%.
f ) **Adverse events (AEs)**
  All participants will be instructed to report any unexpected symptoms or health issues that arise during the study. Adverse events (AEs) will be categorized by severity - mild, moderate, severe, and undesirable (including life-threatening, disabling, or fatal), and assessed for whether they are related to the intervention. A registered Chinese medicine practitioner will document any adverse events at each TCM body constitution assessment. Participants will be encouraged to contact the research team immediately if any undesirable adverse events occur between scheduled visits. Standard operating procedures will be implemented to manage adverse events:
  1) Mild AEs potentially related to the intervention: The drug dosage may be reduced by half, with a follow-up assessment scheduled three days later by a registered Chinese medicine practitioner. If symptoms improve, participants may continue with the reduced dosage, with a possible return to the full dosage based on clinical judgment. If symptoms persist or worsen, the medication will be discontinued.
  2) Moderate AEs potentially linked to the intervention: The medication will be immediately stopped, and a follow-up assessment will occur after three days. If symptoms improve, a reduced dosage may be recommended, with consideration for returning to the full dosage based on clinical judgment. If symptoms do not improve or worsen, the medication will be discontinued.
  3) Severe AEs require immediate discontinuation of the drug. Reintroduction at full dosage will only be considered if the events are determined to be unlikely related to the intervention and once symptoms have fully resolved.
  All details of each adverse event will be recorded, including the nature (intervention-related or not), severity, duration, frequency, and any subsequent actions taken.

**Exploratory Outcomes**

a) **Blood sample**
  The blood sample will be analyzed for hormone levels, inflammatory markers, neurotransmitter levels, and other relevant indicators. The blood test will also include complete blood count and parameters for liver and renal function. Assessment of blood biomarkers will help evaluate the physiological impacts of the intervention objectively.
b) **Urine sample**
  The urine sample will be analyzed for metabolites, markers of oxidative stress, and other relevant indicators. This will involve assessing metabolomic profiles, identifying metabolites related to mental wellness, measuring reactive oxygen species levels, evaluating antioxidant capacity, and examining renal function markers and inflammatory markers.
c) **Fecal sample**
  The fecal sample will be analyzed for microbiome composition, inflammation markers, and other relevant indicators. The analysis will include assessing microbial diversity, identifying microbial species, measuring calprotectin levels as a marker of inflammation, analyzing cytokines, and evaluating digestive functions.
d) **Irritable Bowel Syndrome symptom severity**
  The Irritable Bowel Syndrome-Symptom Severity Score (IBS-SSS) is a validated scale that assesses the severity and frequency of abdominal pain, severity of abdominal distention, dissatisfaction with bowel habits, and interference with quality of life over the past 10 days. Participants will respond to each question on a 100-point visual analogue scale, the range of the total score is 0-500. ^48^
e) **Objective measurement using wearable device**
  Wearable devices will provide objective and quantifiable data on various aspects, including the quality of sleep (sleep duration, sleep latency, and sleep efficiency), and physical activity (calories, step count, and active minutes).
f ) **Body-mind-spirit Well-being Inventory (BMSWBI)**
  The Body-mind-spirit Well-being Inventory (BMSWBI) is a 56-item scale that assesses (1) physical distress, (2) daily functioning, (3) affect, and (4) spirituality. This outcome will only be measured among the level 3 participants.

### 2.7 Participant timeline

**Figure 3.**
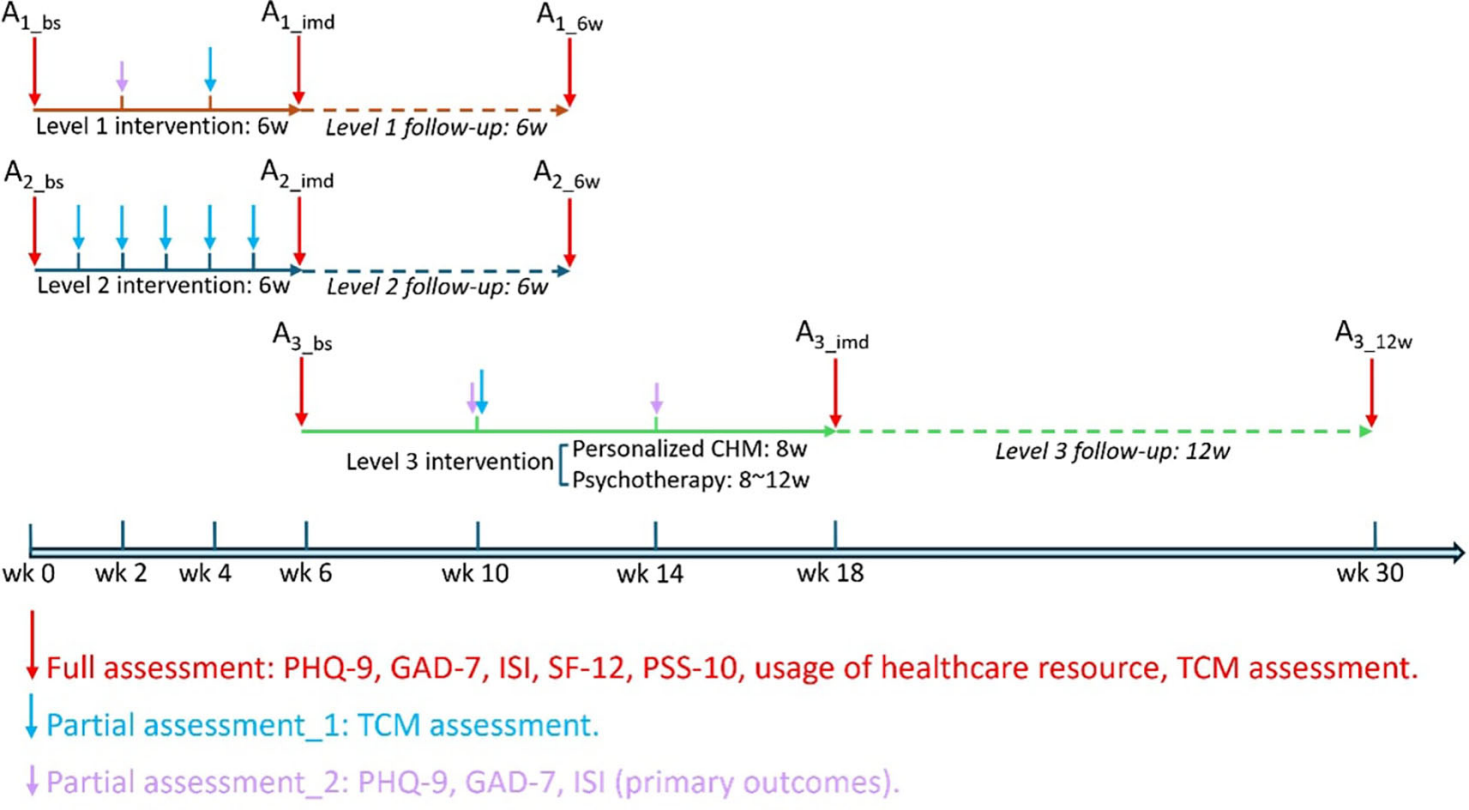
Sketch map of assessments for 3 different levels of intervention. Note: A1_bs: Baseline assessment at level 1 intervention; A1_imd: Assessment immediately after level 1 intervention; A1_6w: Assessment 6 weeks after level 1 intervention; A2_bs: Baseline assessment at level 2 intervention; A2_imd: Assessment immediately after level 2 intervention; A2_6w: Assessment 6 weeks after level 2 intervention; A3_bs: Baseline assessment at level 3 intervention; A3_imd: Assessment immediately after level 3 intervention; A3_12w: Assessment 12 weeks after level 3 intervention. PHQ-9, Patient Health Questionnaire-9; GAD-7, General Anxiety Disorder-7; ISI, Insomnia Severity Index; SF-12, 12-Item Short Form Survey Instrument; PSS-10, Perceived Stress Scale-10; TCM, Traditional Chinese Medicine.

See Table 3 and Figure 3.

**Table 3.** Details of assessments at each level of intervention.

Timepoint	A _1_bs_	A _2_bs_	A _3_bs_	A _1_imd_	A _2_imd_	A _3_imd_	A _1_6w_	A _2_6w_	A _3_12w_
**Enrolment**									
Informed consent	✓	✓	✓						
Demographic data	✓	✓	✓						
Medical history	✓	✓	✓						
Concurrent treatment	✓	✓	✓						
**Primary outcomes**									
PHQ-9	✓	✓	✓	✓	✓	✓	✓	✓	✓
GAD-7	✓	✓	✓	✓	✓	✓	✓	✓	✓
ISI	✓	✓	✓	✓	✓	✓	✓	✓	✓
**Secondary outcomes**									
SF-12	✓	✓	✓	✓	✓	✓	✓	✓	✓
PSS-10	✓	✓	✓	✓	✓	✓	✓	✓	✓
Utilization of healthcare resource	✓	✓	✓	✓	✓	✓	✓	✓	✓
TCM assessment	✓	✓	✓	✓	✓	✓	✓	✓	✓
Compliance rate				✓	✓	✓			
Adverse events				✓	✓	✓			
**Exploratory outcomes**									
Sample collection		✓			✓				
IBS-SSS		✓			✓				
Wearable device		✓			✓				
BMSWBI			✓			✓			✓

Note: A1_bs: Baseline assessment at level 1 intervention; A1_imd: Assessment immediately after level 1 intervention; A1_6w: Assessment 6 weeks after level 1 intervention; A2_bs: Baseline assessment at level 2 intervention; A2_imd: Assessment immediately after level 2 intervention; A2_6w: Assessment 6 weeks after level 2 intervention; A3_bs: Baseline assessment at level 3 intervention; A3_imd: Assessment immediately after level 3 intervention; A3_12w: Assessment 12 weeks after level 3 intervention. PHQ-9, Patient Health Questionnaire-9; GAD-7, General Anxiety Disorder-7; ISI, Insomnia Severity Index; SF-12, 12-Item Short Form Survey Instrument; PSS-10, Perceived Stress Scale-10; TCM, Traditional Chinese Medicine; IBS-SSS, irritable bowel syndrome-symptom severity score; BMSWBI, Body-mind-sprit Well-being Inventory.

### 2.8 Sample size

We first estimated the sample size using the approach routinely employed in the standard randomized controlled superiority trial setting. Estimation of sample size was performed using G*Power 3.1. ^49^ Since there has been no study to evaluate the effectiveness of a stepped-care CHM intervention in patients with mild-to-moderate depression, anxiety, and insomnia, we conservatively estimated the effect size (Cohen’s d) of the intervention on the primary outcome is of small magnitude ~ 0.2. ^50^ Given the two-sided α of 0.05, power of 0.95, the sample size for a standard superiority RCT will be 260. Given the stepped wedge design of the present cluster randomized trial, we multiply the sample size by design effect which equals to 1+(m-1) x ICC, where m is the average cluster size and ICC is the intra-cluster correlation coefficient. ^51^ With reference to previous literature, the ICC equals to 0.01. ^52^ We operationalize the 18 districts in Hong Kong into 4 clusters, therefore the average cluster size m will be 260/4 clusters = 65. Thus, the design effect will be 1+(65-1) x 0.01 = 1.64. Therefore, the total sample size will be 260 x 1.64 = 428 (rounded up from 426.4). Considering the possibility of participant attrition during the 24-week study period, we have intentionally adopted a significant drop-out rate of 30% during the sample size calculation. This decision was made to ensure adequate statistical power. ^53^ As a result, the final sample size was determined to be 612 (428/0.7) participants.

### 2.9 Recruitment

We will approach potential participants in the community via interactive face-to-face campaigns, public activities (e.g., interactive workshops/campaigns) across the territory, and advertisements in local newspapers and social media. Interested individuals will be invited to complete an online assessment to confirm eligibility.

### 2.10 Sequence generation

Randomization of the sequence of initiation in 18 districts will be conducted 4 weeks prior to the commencement of study by an independent researcher (IR) not involved in assessment or delivery of the intervention. All 18 districts will receive no interventions in the first 4 weeks. After the first 4 weeks, 4-6 districts will initiate the stepped-care CMH intervention and this procedure continues at 4-week intervals, until all 18 districts cross over to the intervention phase.

### 2.11 Concealment mechanism

The computer-generated randomized allocation sequence that determines the chronological sequence of initiating the intervention will be securely kept in opaque sealed envelopes by the investigator who will not interact with the participants during recruitment, allocation, or interventions. Project coordinators will contact the IR to retrieve the allocation sequence before the commencement of the intervention. The allocation sequence will be generated by the IR who is not involved in the assessment or delivery of the intervention. This investigator's sole responsibility is to perform randomization in accordance with the study protocol. Screeners will enroll participants, but they will not have access to this list.

### 2.12 Blinding

Participants cannot be blinded due to the nature of the interventions. The research personnel delivering the CHM intervention cannot be blinded either, but they will not be involved in the outcome assessments, which will be carried out by research personnel who are blinded from the study design and the participants' group allocation. Participants will be instructed not to disclose their intervention status to research personnel during the outcome assessments.

### 2.13 Data collection

Following the provision of informed consent during the first visit to the clinic, all participants will complete a validated questionnaire as a baseline assessment. This will be followed by a structured interview to collect basic demographics of each participant, including date of birth, gender, education, employment status, income, household size, and personal habits such as caffeine intake, alcohol consumption, smoking, and exercise routines. Additionally, medical history and any concurrent treatments will be documented. Afterward, participants will undergo a TCM body constitution assessment, during which a Chinese medicine practitioner will prescribe the CHM. At different stages during the intervention period, participants will be required to complete validated questionnaires online and participate in tele-consultations for TCM body constitution assessments (see Table 3 and Figure 3). After completing all interventions, participants will be invited to finish a final assessment at the clinic. A follow-up assessment will take place 6 or 12 weeks after the intervention via tele-consultation. To promote participant retention and ensure complete follow-up, participants in Level 1 will be contacted bi-weekly, and those in Level 2 and 3 will be contacted weekly by phone. One day prior to the face-to-face consultation, we will send participants a message reminder of the appointment date and time. Four attempts will be made on two different days to contact each participant.

### 2.14 Data management

All study data will exclusively be entered electronically within the REDCap system (v14.1.2). Data quality is ensured within the REDCap platform through an integrated script and multiple measures to guarantee data completeness. The extent of actions that each user can undertake is restricted by the rights associated with their respective accounts. To protect participant privacy, sensitive information such as names and phone numbers will be separated from experimental data and securely maintained by designated research personnel. Experimental data will be transferred to an excel spreadsheet, where participants will be de-identified using a coding system. Each code will follow this format: “project name, ^54^ treatment level (I, II, III), and participant identification code.” A unique three-digit number will be assigned to each participant. For example, data from the first participant in the level 1 treatment group will be labeled as JCI001. All study data will be stored on password-protected computers at Hong Kong Baptist University to ensure security and confidentiality. Access to the data will be restricted to project research personnel and the data monitoring committee. Biological samples obtained from participants will be stored in research center, School of Chinese Medicine, Hong Kong Baptist University for 7 years.

### 2.15 Statistical methods

Data will be presented as mean (standard deviation) or frequency (percentage). The Hussey-Hughes model will be utilized for data analysis, where linear mixed models will be used for continuous outcomes, and generalized estimating equations will be used for categorical outcomes. Statistical significance will be considered at p < 0.05. All statistical analyses will be performed using R. All outcomes will be statistically analyzed according to the intention-to-treat ^53^ principle and the per-protocol ^13^ population separately. The process of handling missing data is generally divided into deletion or imputation of missing data. The statistician will use different methods, such as the mean imputation, last observation carried forward, and multiple imputations, to address missing data depending on the specific circumstances.

### 2.16 Oversight and monitoring

A data monitoring committee (DMC) will be established to oversee the collection, storage, and analysis of data throughout the trial. The DMC will comprise the principal investigator (ZXB), two co-investigators (DJY and QL), and an independent principal investigator who is not affiliated with the project. This diverse composition ensures both accountability and impartiality in monitoring the study's progress. The committee will convene bimonthly to review data integrity, participant safety, and adherence to study protocols. In addition, the DMC will address any challenges or issues that arise during the trial, ensuring that appropriate measures are taken to maintain the trial’s scientific rigor and ethical standards.

### 2.17 Protocol amendment

Amendment protocol modifications, such as changes to eligibility criteria, outcomes, or dosage adjustments, will first be discussed among the research team to reach consensus. Following this, the research ethics committee of Hong Kong Baptist University will be informed, and an amendment form will be submitted for approval. Once approved, the research team will update the trial registration record and begin implementing the new procedures.

### 2.18 Dissemination plans

Any results from this trial (publications, conference presentations) will be published in peer-reviewed journals and conference proceedings. Upon reasonable request, the corresponding author can provide complete protocol information as well as relevant data.

## 3. Discussion

The existing literature indicates that TCM, especially CHM is underutilized in the treatment of sleep and mental disorders, ^26^ despite its promising therapeutic effects for alleviating depression, anxiety and insomnia. Given the presence of over ten thousand Chinese medicine practitioners in Hong Kong and the region's critical shortage of mental health professionals, TCM could greatly bolster mental health service provision. ^55^ Moreover, TCM employs a holistic approach that targets not only psychological but also physiological dimensions, making it a comprehensive option for healthcare. This holistic approach is generally subjected to less stigma than conventional mental health treatments, thereby encouraging individuals to seek necessary care without fear of being labeled. More importantly, several clinical trials have demonstrated the efficacy of TCM in alleviating symptoms associated with mental and sleep disorders, often with fewer side effects than those observed with conventional treatments. ^30– 33, 56^ These characteristics underscore the substantial potential for a broader incorporation of TCM into mental healthcare systems for managing depression, anxiety and insomnia.

To effectively evaluate the integration of TCM into the mental healthcare system, the stepped-wedge design, which is commonly used for evaluating service delivery or policy interventions at the cluster level, will be adopted in the present study. ^37^ This design is particularly powerful when substantial cluster-level effects are present or if the clusters are large, as it can provide higher statistical power than a parallel design. ^37, 57^ The randomization within the stepped wedge design helps avoid allocation bias, allowing for a more robust assessment of the effectiveness of the intervention. ^58^ Additionally, this design also offers the opportunity to measure the time effect of the intervention. ^37^ Furthermore, the stepped wedge design is more ethical as it does not withhold the intervention from any participants. ^36^ This design also addresses logistical and practical considerations more effectively when the intervention can only be implemented in stages. This staged implementation helps alleviate the challenges posed by limited resources, ^36^ making the stepped wedge design a both financially and logistically feasible option for large-scale evaluations.

To enhance the real-world applicability of this trial, we will employ a stepped care model, which some countries have already adopted in their care guidelines for mental disorders. ^16, 59^ Stepped care model is recognized for its cost-effectiveness, ^42, 60^ starting with the least restrictive interventions, such as herbal tisanes. These initial measures minimize both costs and personal inconvenience for patients, while also requiring less specialist time, thereby reducing overall healthcare costs. In the stepped-care framework, more advanced treatments are generally reserved for participants who do not benefit from simpler first-line treatments. This approach optimizes the utilization of healthcare resources by escalating from standardized herbal formula treatments to personalized treatments and psychotherapy as needed.

## 4. Conclusion

This study is designed to rigorously evaluate the effectiveness of a stepped care CHM intervention within a real-world setting, employing a methodologically robust stepped-wedge design for patients experiencing mild to moderate depression, anxiety, and insomnia. The findings will inform future decision-making for integrating CHM into mainstream healthcare systems in Hong Kong, China.

### Protocol version

Protocol version: 20240911-001; date: September 11, 2024.

## Ethical approval and consent

The study was approved by the Hong Kong Baptist University Research Ethics Committee (Approval No.: REC/22-23/0709) on April 8, 2024, and prospectively registered with the Chinese Clinical Trial Registry (ChiCTR2400083685) on April 30, 2024. Recruitment started on May 1, 2024, and has already been completed. Data collection is expected to end in July 2025).

Before participation, verbal and written information on the study will be provided. All individuals will undergo a detailed informed consent process where they are informed about the study’s procedures, risks, and benefits, ensuring their right to withdraw at any time. Written informed consent will be obtained before baseline outcome assessments.

## Consent for publication

The authors affirm that all individuals involved in this study have provided their informed consent for the publication of the data and associated research findings.

## Data Availability

No data associated with this article. Upon completion of the project, the results will be published in peer-reviewed journals, presented at international conferences, and shared with the community through local press conferences. Figshare: SPIRIT Checklist: Study Protocol for a Stepped-Wedge Cluster Randomized Trial on the Effectiveness of Chinese Herbal Medicine in Improving Sleep and Mental Health in Adults. https://doi.org/10.6084/m9.figshare.29062862. ^61^ Data are available under the terms of the Creative Commons Attribution 4.0 International license (CC-BY 4.0).
