# Supplementary material for: Effectiveness of Chinese herbal medicine on improving sleep and mental health in adults: Study protocol for a stepped-wedge cluster randomized trial
Source: F1000Res. 2025 Oct 13;14:556. Originally published 2025 Jun 4. [Version 2] doi: 10.12688/f1000research.163698.2 (PMC12536410; doi:10.12688/f1000research.163698.2)
Supplement: Peer review history

# Reviewer report: Reviewer response for version 2

**DOI:** 10.5256/f1000research.189444.r423284

**Published:** 2025-10-16

**Authors:** Yujin Choi

**Affiliations:** 1 Korea Institute of Oriental Medicine, Daejeon, South Korea

The authors have adequately addressed the concerns raised in the initial review.

Is the study design appropriate for the research question?

Yes

Is the rationale for, and objectives of, the study clearly described?

Yes

Are sufficient details of the methods provided to allow replication by others?

Yes

Are the datasets clearly presented in a useable and accessible format?

Not applicable

Reviewer Expertise:

Herbal medicine, Acupuncture, Neuropsychiatry, Clinical trial, Systematic review

I confirm that I have read this submission and believe that I have an appropriate level of expertise to confirm that it is of an acceptable scientific standard.

# Reviewer report: Reviewer response for version 1

**DOI:** 10.5256/f1000research.180090.r409017

**Published:** 2025-09-04

**Authors:** Yujin Choi

**Affiliations:** 1 Korea Institute of Oriental Medicine, Daejeon, South Korea

This manuscript presents a study protocol for a multicenter, stepped-wedge cluster randomized trial evaluating the effectiveness of a stepped-care Chinese herbal medicine (CHM) intervention for adults with mild-to-moderate depression, anxiety, and insomnia. This represents an interesting research plan, and the protocol is generally well-written, though there are several areas that would benefit from clarification or refinement.

**Major issues**

**1. Multiple primary outcomes without statistical plan**

 The protocol designates three separate conditions (depression, anxiety, insomnia) as primary outcomes without specifying a clear statistical analysis plan for handling multiple primary comparisons. This approach may lead to Type I error inflation. The authors might consider either: (1) selecting a single primary outcome with the others as secondary, (2) implementing hierarchical testing procedures, or (3) applying appropriate multiplicity adjustments (e.g., Bonferroni correction). Clarification of the statistical approach for multiple primary endpoints would strengthen the protocol.

**2. Blinding strategy for patient-reported outcomes**

 The protocol states that "research personnel who are blinded from the study design and the participants' group allocation" will conduct outcome assessments, and "participants will be instructed not to disclose their intervention status." However, it appears that all primary outcomes (PHQ-9, GAD-7, ISI) are self-administered questionnaires completed by participants themselves. Given that participants cannot be blinded to their intervention status due to the nature of CHM treatment, this may present limitations for the blinding strategy.

**Minor issues**

**Consent for publication**

 Consider stating "Not applicable" rather than the current statement, as this protocol may not contain individual patient data requiring specific consent for publication

Is the study design appropriate for the research question?

Yes

Is the rationale for, and objectives of, the study clearly described?

Yes

Are sufficient details of the methods provided to allow replication by others?

Yes

Are the datasets clearly presented in a useable and accessible format?

Not applicable

Reviewer Expertise:

Herbal medicine, Acupuncture, Neuropsychiatry, Clinical trial, Systematic review

I confirm that I have read this submission and believe that I have an appropriate level of expertise to confirm that it is of an acceptable scientific standard, however I have significant reservations, as outlined above.

# Response

**Published:** 2025-10-07

**Authors:** Hiu To Tang

**Affiliations:** Hong Kong Baptist University, Hong Kong, Hong Kong

Dear Dr Choi,

 Thank you for your time and for the thorough reading of our manuscript. We are grateful for the insightful and favorable comments. We have revised the manuscript taking into consideration all the feedback received. For clarity, the reviewers’ comments are presented in bold, our responses are in regular font, and the corresponding changes in the manuscript are in italic. We believe these revisions have significantly strengthened clarity and methodological rigor. We hope we have addressed all the concerns and that the revised manuscript is now suitable for publication in F1000Research. Thank you for your time and consideration.

 Yours sincerely,

 Tang Hiu To

**This manuscript presents a study protocol for a multicenter, stepped-wedge cluster randomized trial evaluating the effectiveness of a stepped-care Chinese herbal medicine (CHM) intervention for adults with mild-to-moderate depression, anxiety, and insomnia. This represents an interesting research plan, and the protocol is generally well-written, though there are several areas that would benefit from clarification or refinement.**

**Major issues**

**1. Multiple primary outcomes without statistical plan**

**The protocol designates three separate conditions (depression, anxiety, insomnia) as primary outcomes without specifying a clear statistical analysis plan for handling multiple primary comparisons. This approach may lead to Type I error inflation. The authors might consider either: (1) selecting a single primary outcome with the others as secondary, (2) implementing hierarchical testing procedures, or (3) applying appropriate multiplicity adjustments (e.g., Bonferroni correction). Clarification of the statistical approach for multiple primary endpoints would strengthen the protocol.**

**Response:** We thank the reviewer for this important suggestion. Holm procedure will be performed to control the Type I error inflation. The statistical methods section (2.15) has been revised accordingly.

***Revised manuscript:*** *Data will be presented as mean (standard deviation) or frequency (percentage). The Hussey-Hughes model will be utilized for data analysis, where linear mixed models will be used for continuous outcomes, and generalized estimating equations will be used for categorical outcomes. Pairwise comparisons will be performed using linear contrast. Statistical significance will be considered at p < 0.05. The Holm procedure will be applied to address multiplicity. All statistical analyses will be performed using SAS OnDemand for Academics. All outcomes will be statistically analyzed according to the intention-to-treat principle and the per-protocol population separately.*

**2. Blinding strategy for patient-reported outcomes**

**The protocol states that "research personnel who are blinded from the study design and the participants' group allocation" will conduct outcome assessments, and "participants will be instructed not to disclose their intervention status." However, it appears that all primary outcomes (PHQ-9, GAD-7, ISI) are self-administered questionnaires completed by participants themselves. Given that participants cannot be blinded to their intervention status due to the nature of CHM treatment, this may present limitations for the blinding strategy.**

**Response:** Thank you for your insightful feedback on the blinding strategy. We acknowledge the limitation that participants cannot be blinded to their intervention, which is an important consideration when interpreting results from self-rated questionnaires. To strengthen our methodology and complement these subjective outcomes, we have incorporated clinician-rated assessments, such as the Traditional Chinese Medicine body constitution assessment, along with objective evaluation methods, including biological samples and wearable devices. This approach aims to provide a more robust and multi-faceted analysis of the intervention's effects.

***Revised manuscript:***

 Blinding of participants is not feasible for this trial due to the nature of the interventions. The research personnel responsible for delivering the CHM intervention cannot be blinded either. To mitigate potential bias, the clinician-rated TCM body constitution assessment and all objective outcomes, including data from wearable devices and the collection of biological samples, will be conducted by research personnel who are blinded to both the study design and participant group allocation. These assessors will have no role in delivering the intervention, and all participants will be instructed to conceal their allocation status during these assessments.

**Minor issues**

**Consent for publication**

**Consider stating "Not applicable" rather than the current statement, as this protocol may not contain individual patient data requiring specific consent for publication**

**Response:** Thank you for the suggestion. We have updated the "Consent for publication" section to "Not applicable" as recommended.

# Reviewer report: Reviewer response for version 1

**DOI:** 10.5256/f1000research.180090.r390086

**Published:** 2025-08-14

**Authors:** Arshiya Sultana

**Affiliations:** 1 National Institute of Unani Medicine, Bangalore, Karnataka, India

**Thanks for inviting me to review this manuscript.**

**Manuscript Evaluation**

 The submitted manuscript addresses a relevant and novel topic of interest to the journal’s readership. No revisions are required and can be considered for indexing. The authors have carefully address the following concerns

**Minor Comments**

1. **Title:** The current title accurately represents the overall scope of the study. By structuring it according to the PICOS framework (Population, Intervention, Comparison, Outcomes, Study Design), the authors have enhanced both the transparency and methodological rigor of the research.

 

1. **Abstract:** Abstract is structured.
2. **Introduction: **The background and rationale are well-articulated. However, the authors clearly stated the study's objectives and outlining its contribution but it is mentioned in methodology which have to shifted to introduction part. To further enhance clarity, the authors could consider including a structured illustration or flow diagram.

 

1. **Methodology: **The methodology is clearly presented.
2. **Discussion and Conclusion: **It is meticulously written
3. **References: **Recent and high-quality references are incorporated to strengthen the scientific foundation and relevance of the review. However, few references are very old more than five years. The manuscript does include self-citations.
4. **General Comments:**

The manuscript is clear and relevant to the field.

 The authors are advised to review the manuscript for potential instances of plagiarism and ensure proper citation of all sources.

 The ethics statement is written.

 The data availability statements are mentioned.

Is the study design appropriate for the research question?

Yes

Is the rationale for, and objectives of, the study clearly described?

Yes

Are sufficient details of the methods provided to allow replication by others?

Yes

Are the datasets clearly presented in a useable and accessible format?

Yes

Reviewer Expertise:

Gynaecology and obstetrics, mental health in women, urogynecology, Herbal medicine

I confirm that I have read this submission and believe that I have an appropriate level of expertise to confirm that it is of an acceptable scientific standard.
